# Enhancing Wetland Mapping: Integrating Sentinel-1/2, GEDI Data, and Google Earth Engine

**DOI:** 10.3390/s24051651

**Published:** 2024-03-03

**Authors:** Hamid Jafarzadeh, Masoud Mahdianpari, Eric W. Gill, Fariba Mohammadimanesh

**Affiliations:** 1 Department of Electrical and Computer Engineering, Memorial University of Newfoundland, St. John’s, NL A1B 3X5, Canada; hjafarzadeh@mun.ca (H.J.); m.mahdianpari@mun.ca (M.M.); ewgill@mun.ca (E.W.G.); 2 C-CORE, St. John’s, NL A1B 3X5, Canada

**Keywords:** classification, GEDI, Google Earth Engine, LiDAR, Sentinel, wetland

## Abstract

Wetlands are amongst Earth’s most dynamic and complex ecological resources, serving productive and biodiverse ecosystems. Enhancing the quality of wetland mapping through Earth observation (EO) data is essential for improving effective management and conservation practices. However, the achievement of reliable and accurate wetland mapping faces challenges due to the heterogeneous and fragmented landscape of wetlands, along with spectral similarities among different wetland classes. The present study aims to produce advanced 10 m spatial resolution wetland classification maps for four pilot sites on the Island of Newfoundland in Canada. Employing a comprehensive and multidisciplinary approach, this research leverages the synergistic use of optical, synthetic aperture radar (SAR), and light detection and ranging (LiDAR) data. It focuses on ecological and hydrological interpretation using multi-source and multi-sensor EO data to evaluate their effectiveness in identifying wetland classes. The diverse data sources include Sentinel-1 and -2 satellite imagery, Global Ecosystem Dynamics Investigation (GEDI) LiDAR footprints, the Multi-Error-Removed Improved-Terrain (MERIT) Hydro dataset, and the European ReAnalysis (ERA5) dataset. Elevation data and topographical derivatives, such as slope and aspect, were also included in the analysis. The study evaluates the added value of incorporating these new data sources into wetland mapping. Using the Google Earth Engine (GEE) platform and the Random Forest (RF) model, two main objectives are pursued: (1) integrating the GEDI LiDAR footprint heights with multi-source datasets to generate a 10 m vegetation canopy height (VCH) map and (2) seeking to enhance wetland mapping by utilizing the VCH map as an input predictor. Results highlight the significant role of the VCH variable derived from GEDI samples in enhancing wetland classification accuracy, as it provides a vertical profile of vegetation. Accordingly, VCH reached the highest accuracy with a coefficient of determination ($R^{2}$) of 0.69, a root-mean-square error (RMSE) of 1.51 m, and a mean absolute error (MAE) of 1.26 m. Leveraging VCH in the classification procedure improved the accuracy, with a maximum overall accuracy of 93.45%, a kappa coefficient of 0.92, and an F1 score of 0.88. This study underscores the importance of multi-source and multi-sensor approaches incorporating diverse EO data to address various factors for effective wetland mapping. The results are expected to benefit future wetland mapping studies.

## 1. Introduction

Wetlands are highly productive, yet they are the most vulnerable ecosystems, straddling terrestrial and aquatic systems [1,2]. Due to their ecological richness, multifunctionality, and conservation concerns, wetlands are often referred to as “the kidney of the Earth” [3] and have drawn the attention of scientific communities. They offer a diverse range of services that contribute to human well-being and nature, serving as protective buffers against flood and storm damage, improving water quality, promoting aquatic and plant biomass productivity, stabilizing shorelines, aiding in plant collection and retaining contaminants [4]. However, wetlands face degradation in terms of coverage and biodiversity due to natural and human-induced disturbances, such as global warming, rising sea levels, and agricultural activities [5,6]. As such, accurate mapping and consistent monitoring are crucial for managing and conserving wetland resources, protecting them from degradation, evaluating restoration efforts and ensuring future sustainability [7,8].

With a view toward mitigating the existing environmental and biodiversity crises, global biodiversity changes can be monitored using several measures referred to as essential biodiversity variables (EBVs) [9]. EBVs function as a health indicator of an ecosystem and can be defined as metrics reporting on ecosystem dynamics and biodiversity. Ecological traits, species populations, diverse community compositions, as well as ecosystem function and structure are some of the more salient EBVs related to wetlands. Most of these variables can be inferred, if not measured directly, through remote sensing techniques. In general, the utility of remote sensing in characterizing EBVs and biodiversity is being increasingly acknowledged. Remote sensing data can provide insight into some EBVs; thus, it is important to accurately link EBVs with remote sensing data to facilitate long-term global monitoring [10]. Within this framework, vegetation canopy height (VCH) and structure hold great potential in developing EBVs for wetland landscapes. Launching these concepts (e.g., VCH) acts as a new toolkit for assessing the state of wetland realms and determining effective action toward monitoring tasks. VCH provides a perspective of the vertical dimension of vegetation, offering valuable information on habitat diversity, species richness assessment, and ecosystem resilience to environmental changes [11,12]. Furthermore, the quantitative assessment of wetland vegetation characteristics, including canopy height and vegetation cover fraction, contributes crucial insights for effective ecosystem management practices [13,14].

The difficulty of accessing wetlands and their heterogeneous nature pose significant challenges for classifying and assessing their cover types, both spatially and temporally [15,16]. Inexpensive remote sensing data offer synoptic, repeated, and reliable observations, making them favorable for monitoring the natural environment [1]. The integration of multiple sensors and measurements, such as optical and Synthetic Aperture Radar (SAR), enables space-based remote sensing systems to perform large-scale assessments of vegetation cover and biomass, surpassing the capabilities of traditional ground-based field measurements. However, relying solely on optical or SAR datasets tends to overlook some vegetation properties and structures due to the uncertainties inherent in the data sources.

So far, numerous studies have been carried out utilizing multi-source remotely sensed data to map, monitor, and assess the global wetlands from local to sub-continental scales [16,17]. These studies have primarily focused on SAR and optical imagery [18,19]. In contrast, the current study goes beyond the existing approaches by exploring additional features contributing to comprehensive wetland characterizations. We recognize that wetlands exhibit diverse forms and can be characterized in multiple ways, necessitating the consideration of a broader range of features.

In this context, the present work explores new aspects in terms of using multi-source and multi-sensor data. It evaluates the ability of different Earth observation (EO) datasets for wetland classification, focusing on NASA’s Global Ecosystem Dynamics Investigation (GEDI) LiDAR data. Taking these capabilities into account has the potential to boost the remote sensing toolbox for wetland habitat monitoring by adding LiDAR-derived VCH, which includes the vertical structure of vegetation and additionally provides information complementary to that obtained from optical and SAR imagery. Incorporating supplementary data can offer a unique understanding of wetland characteristics beyond responses achieved with conventional remote sensing imagery, including features such as vegetation height, density, biomass distribution, etc.

Previous research efforts have illustrated the potential of integrating space-borne LiDAR—particularly GEDI—with optical and SAR imagery to conduct a detailed assessment of canopy height and biomass. Due to limited sampling and spatial resolution of grids, LiDAR measurements rely on either accepting sampling errors using statistical methods or integrating them with other remote sensing data. This allows the leveraging of accurate but sparse LiDAR data to calibrate less accurate continuous datasets (compared to full-cover LiDAR products). In [20], Qi et al. improved forest height modeling by incorporating simulated GEDI LiDAR data and TanDEM-X InSAR data in sites located in the United States and Costa Rica. In addition, canopy height mapping for Chinese forests was achieved by Liu et al. through the integration of GEDI and ICESat-2 data [21]. Dorado-Roda et al. integrated airborne LiDAR and GEDI data in their study [22], estimating the canopy height maps and biomass in Mediterranean forests. Furthermore, biomass estimation was explored in [23] over Sonoma County in California, USA, leveraging simulated data from three missions: GEDI, ICESat-2, and the NASA–Indian Space Research Organization (ISRO) SAR (NISAR).

Building on this, advancements in cloud-based computing resources and open-access data policies for high-resolution EO datasets have revolutionized large-scale wetland monitoring [24]. The availability of datasets, particularly from Landsat and Sentinel archives, has enhanced the understanding of wetland ecosystems, facilitating effective management and conservation [25]. Google Earth Engine (GEE) exemplifies this innovative technology by providing access to vast amounts of satellite data and a web-based workbench environment for geospatial data analysis, incorporating image processing and machine learning (ML) algorithms [6,26]. Its parallel processing architecture enables the storage and analysis of a massive volume of geospatial data that speed up the mapping and monitoring tasks [27], supporting diverse applications [28,29].

This study seeks to enhance the accuracy of wetland mapping by integrating free-access EO data sources in the GEE cloud-computing environment. Despite the scientific accomplishments and the availability of various data sources, a knowledge gap exists in the application of remotely sensed data for VCH at the species level across different wetland ecosystems. At its core, this study evaluates the potential of footprint space-borne LiDAR samples in estimating gridded VCH within a unique Newfoundland ecosystem across four pilot sites and determines the effect of the VCH as an input predictor for accurate wetland mapping.

In summary, this study pursues the following main objectives:

- Firstly, it establishes an ML-based approach to integrate GEDI footprints with multi-source EO data to generate VCH maps for wetland regions using GEE. The efficiency of GEDI-derived canopy height is also assessed under diverse terrain and land cover conditions distributed across the study sites. This integration focuses on exploring the potential of combining space-borne LiDAR point samples from GEDI footprints to generate VCH maps.
- Secondly, the study utilizes the combined VCH dataset and multi-source EO data to produce wetland classification maps. This step is crucial for investigating the importance of VCH to achieving enhanced wetland mapping.

Section 2 and Section 3 provide a comprehensive overview of the study sites, multi-source and multi-sensor datasets, and the applied methodology. The results are presented in Section 4, and the significance of the employed methodology is discussed in Section 5. Finally, Section 6 draws the conclusions.

## 2. Study Sites and Datasets

### 2.1. Study Sites

**Figure 1:**
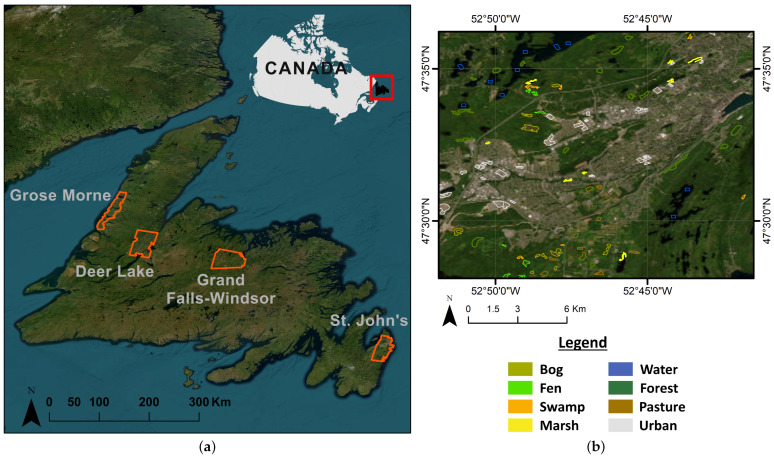
(**a**) An overview of the Island of Newfoundland and pilot sites; (**b**) a representation of collected field data in St. John’s area.

To apply the proposed workflow, we chose four sub-provincial scale sites on the Island of Newfoundland, Canada, as study areas (see Figure 1). The total area covered by satellite imagery in these pilot sites is about 4229 km^2^. The predominant land cover types are categorized into two general groups: (1) wetlands (i.e., bog, fen, swamp, and marsh) and (2) non-wetlands (i.e., deep water, forest, pasture, urban). The non-wetland areas are also of interest due to their potential as areas for development or land use change.

The first pilot site, St. John’s City in the Avalon Peninsula area, situated in the southeast within the Maritime Barren eco-region, encounters an oceanic climate characterized by misty, cool summers and relatively temperate winters. Both Grand Falls-Windsor in the north-central region and Deer Lake in the northern area, designated as the second and third pilot sites, lie within the Central Newfoundland eco-region. These areas exhibit a continental climate characterized by cool summers and cold winters. On the far west coast in the Northern Peninsula eco-region, our fourth pilot site, Gros Morne, experiences a maritime-type climate with cool summers and mild winters [4].

These pilot sites represent the diverse regional characteristics of the landscape and vegetation across the island, with varying latitudes, longitudes, elevations, slopes, canopy heights, and canopy covers. They feature a range of geological formations, vegetation types, and climate conditions [6], allowing for a comparative analysis of the GEDI mission and its integration with other data sources. Detailed information on each pilot site can be found in Table 1.

### 2.2. Reference Data Repository

Wetland reference data collected from field campaigns are a valuable resource for geoscientists conducting research in the field of wetland ecology and management. It provides a wealth of information that can be utilized for various objectives, such as training and testing models for wetland identification and mapping, understanding the ecological characteristics of wetlands in a specific region, and tracking changes in wetland conditions over time. In this study, the reference samples of wetland/non-wetland classes were collected via field campaigns by multiple organizations. These campaigns involved visiting, identifying, and collecting data from all mentioned pilot sites (see Figure 1).

A size-based sorting approach was implemented for in-situ recorded reference polygons to enhance the accuracy and assessment robustness. This involved categorizing polygons based on their size and subsequently allocating them to either training or testing sets alternately. This method guaranteed the independence of the samples used to generate the training and testing polygons. Furthermore, the alternative assignment strategy aimed to achieve a balanced distribution, with ≈50% of polygons assigned to each group. The balanced allocation ensured that both the training and testing sets consisted of an equal number of small and large polygons. By doing so, the pixel counts were kept similar, effectively addressing the substantial variations in intra-wetland sizes. Table 2 indicates the total number of reference polygons assigned for each land cover type.

### 2.3. Multi-Source/Multi-Sensor Data Collection on GEE and Pre-Processing

This study introduced and utilized a combination of multi-source EO data for classification, thereby fusing the strengths inherent in each dataset and enhancing the ability to discriminate different wetland classes. These include Sentinel-1 and -2 imagery, point-based height measurements from GEDI, the Merit Hydro dataset, ERA5, and elevation data with topographical derivatives. It is hypothesized that, with additional information, wetland areas can be identified and classified based on surface structure and hydrologic characteristics that may otherwise be indistinguishable in optical or SAR data. The GEE platform was used to acquire and pre-process all datasets. The subsequent sections provide a detailed overview of each data source.

#### 2.3.1. SAR and Optical Data

This study utilized the European Space Agency’s (ESA) Sentinel-1 C-band (≈5.5 cm wavelength) SAR imagery, which offers all-weather and all-time observations [30]. Our analysis considered the backscattering coefficient products derived from dual-band cross-polarization VV + VH, with a pixel size of 10 m. A composite summer image was generated by calculating the mean values of backscattering data spanning from June to September 2021 (four months). This approach is employed to mitigate the impact of speckle noise inherent in SAR images [31].

In addition, ESA’s Sentinel-2 optical multispectral imagery, a constellation of a pair of identical satellites, was utilized. These satellites collectively cover the Earth’s surface every five days, providing imagery with 10 m, 20 m, and 60 m spatial resolution across 13 spectral bands [30]. A summer composite of the Sentinel-2 surface reflectance products was generated by employing the four-month extraction of Sentinel-2 observations and determining the median values across all pixels. This approach effectively helped to reduce the effects of cloudy and other unwanted pixels [31].

Further, to match the pixel size of Sentinel-1, the Sentinel-2 composite was upsampled to a 10 m pixel size for all spectral bands.

#### 2.3.2. GEDI Relative Height (RH) Data

Since its launch in December 2018, the GEDI instrument has revolutionized the study of vegetation structure. It is a laser altimeter with a full-waveform footprint dedicated exclusively to collecting unique data on the height and density of vegetation [32]. The GEDI data available in the GEE environment are created by selecting a subset of variables from NASA’s GEDI level 2A (L2A) Global Footprint-Level Elevation and the Height Metrics Product [33].

**Figure 2:**
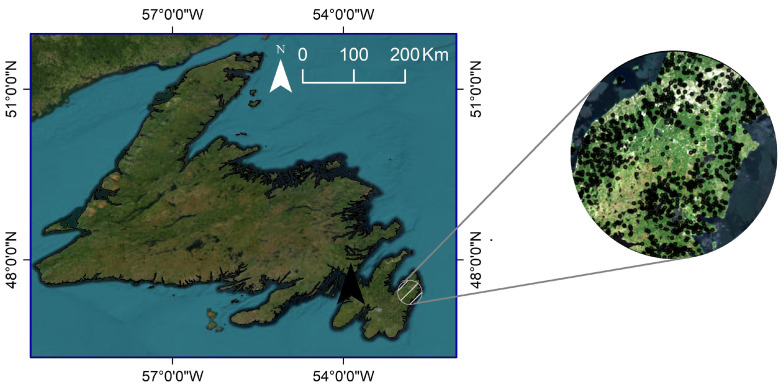
A visual example of the distribution of GEDI RH95 points on GEE in the St. John’s site.

The relevant variables of the product to be used in the current study are relative vegetation canopy height metrics (ranging from RH0 to RH100), capturing the height above the ground. For instance, RH50 represents the height of medium energy returns, while RH100 specifies the top of the canopy height. As the RH100 is associated with the first returned pulse and depends on signal-to-noise ratio (SNR) measurements, it may contain noise [34], leading to inaccurate estimates in complex canopy areas. Comparisons with GEDI-simulated waveforms indicate that RH95 is the most reliable estimate of canopy height [35], while RH100 consistently underestimates canopy heights. Hence, RH95 was employed as the vegetation stand height for the wetland to forest covers in this work, and was utilized as reference data for training and testing the prediction model. Remarkably, GEDI footprints have a diameter of 25 m and are spaced 60 m apart along a ground track [36]. A representation of GEDI footprints in the St. John’s area is illustrated in Figure 2. Note that the GEDI observations for each pilot site were split into two training and testing parts with 70% and 30% ratios. The details on GEDI training and testing dataset quantity are presented in Table 3.

GEDI LiDAR observations may include outliers caused by factors such as topography, photon return, and atmospheric conditions [37]. Here, the RH metric data are refined by defining stricter screening conditions to exclude the outliers and provide good-quality GEDI data collection. The following processing steps are involved in data filtering:

- Filtering based on a quality flag parameter is performed on GEDI observations, removing measurements with a quality flag value of zero. A value of 1 indicates a valid waveform, while a value of 0 indicates an invalid waveform.
- The ESA has developed a comprehensive global land cover map at a spatial resolution of 10 m, utilizing Sentinel-1 and -2 datasets [38]. This map, known as ESA WorldCover V100, delineates 11 distinct land cover classes based on the classification system of the United Nations (UN) Food and Agriculture Organization (FAO). These classes include tree cover, shrubland, grassland, cropland, build-up, bare/sparse vegetation, snow-ice, permanent water bodies, herbaceous wetland, mangroves, and moss-lichen. Our study utilized the ESA land cover map to mask out non-vegetated regions in VCH maps.
- GEDI points were further screened using a defined threshold and multiple model-runs. Accordingly, the average canopy height was considered as the threshold for filtering GEDI RH metric data, calculated based on the GEDI-V27 product—a global canopy height map available from GEE with a 30 m spatial resolution [39]. GEDI points with a height value greater than twice the determined threshold at each pilot site were excluded from the analysis.

#### 2.3.3. Merit Hydro

The Multi-Error-Removed Improved-Terrain (MERIT) Hydro dataset presents a global flow direction map at 3-arc-sec resolution. This map is derived from the MERIT digital elevation model (DEM) and multiple water body datasets [40]. Despite being recently released, this dataset is increasingly being used for hydrological applications, including the global reconstruction of river flows [41] and the modeling of floodplain inundation dynamics [42]. Within this study, we incorporated two parameters derived from the MERIT Hydro dataset: (1) flow accumulation parameter [43] and (2) height above nearest drainage (HAND) parameter [44]. The HAND parameter represents the elevation difference between a pixel in a DEM and the nearest drainage network, while flow accumulation quantifies the cumulative weight of all pixels flowing into each downslope pixel. The inclusion of these parameters in wetland mapping is motivated by the close correlation between wetland ecosystems, topography, and hydrology, thereby introducing novel aspects to the analysis.

#### 2.3.4. ERA5

The supplementary climate data utilized in this study were acquired from the global ERA5 dataset, the fifth-generation European ReAnalysis [45]. It supplies global hourly measurements of various atmospheric and weather-related variables such as temperature, precipitation, wind, and pressure. It is accessible in a regular latitude–longitude grid with 31 km spatial resolution. In this work, temperature and precipitation data were collected from ERA5 at a spatial resolution of 30 km, and the average monthly temperature and precipitation were calculated.

#### 2.3.5. SRTM-DEM

The 30 m (1-arc-second) Shuttle Radar Topography Mission (SRTM) DEM [46] was originally designed by NASA to deliver the first consistent and relatively high-quality elevation data on a near-global scale [47]. The SRTM-DEM (V4), available in the GEE database, is the reprocessed version that addresses data voids and facilitates its ease of use.

Besides the elevation, both aspect and slope parameters were computed and integrated into our analysis. These terrain-related variables contribute valuable information for understanding the topographical characteristics of the pilot sites, enabling comprehensive interpretation of the wetland environments under investigation.

Considering all the abovementioned geospatial data sources, different features were extracted (listed in Table 4). Each variable was selected in accordance with its unique ability to capture wetland information.

## 3. Methods

### 3.1. Methodology Overview

**Figure 3:**
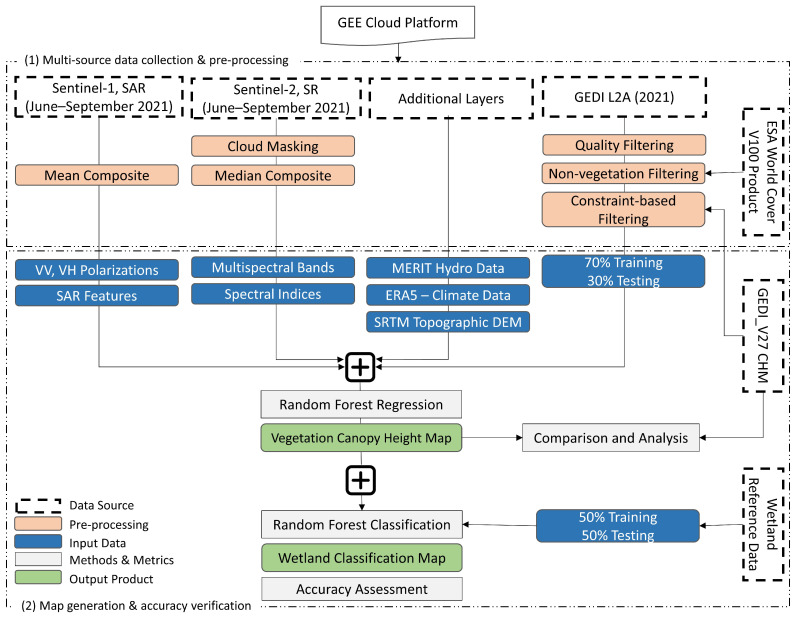
A representation of the approach developed for VCH and wetland mapping utilizing multi-source and multi-sensor remote sensing data.

Accurate estimation of VCH is essential for understanding ecosystem dynamics and biodiversity. To achieve this, we developed a process flow diagram outlining the different research stages. Figure 3 depicts the process flow diagram for the present study. The first phase involves data acquisition and pre-processing, followed by extracting various features. In the second phase, VCH and classification maps are obtained and assessed. The adopted approach relies on only publicly and freely accessible datasets that are widely available, allowing for easy adaptation to other heterogeneous wetland settings. Detailed overviews of each stage are provided in the following subsections.

### 3.2. Vegetation Canopy Height (VCH) Model

While several ML algorithms are available in the literature, Random Forest (RF), as a good ‘first-choice algorithm’, is proving to be a fairly accurate, computationally efficient, and easy-to-use ML algorithm for classification and regression compared to other methods [48]. RF is a versatile and robust method capable of handling the complex non-linear relations between the input and target variables [49]. It requires less data setup and model parameterization and makes fewer assumptions, making it suitable for high-dimensional datasets. The bootstrapping technique and parallel computing in RF reduce prediction error and accelerate the process [50]. Importantly, the RF algorithm offers a mechanism for estimating variable importance, enabling researchers to identify the most influential remote sensing features for accurate mapping.

Despite being able to penetrate the canopy to reach the ground and measure the height of the canopy, there are spatial discontinuities in GEDI footprints across the Earth’s surface. The ancillary optical or SAR remote sensing datasets are often integrated to generate spatially continuous and consistent canopy height maps [32]. However, it should be noted that the method for predicting canopy height may not always be accurate due to factors such as the saturation of spectral reflectance in complex vegetation structures. This study includes additional important features in interpolating GEDI points to overcome such issues in retrieving canopy height and making the prediction model more stable.

As listed in Table 4, the inputs to the RF regression model consisted of 30 independent variables: 18 optical surface reflectance bands and spectral indices; 5 SAR-derived features; 2 MERIT Hydrography bands; 2 ERA5–Climate bands; and 3 SRTM DEM-derived features. A stacked GeoTiff image was exported with these multi-source metrics stored as separate bands. By incorporating these diverse inputs, the RF approach interpolated the pre-processed and filtered LiDAR footprints from space-borne GEDI into a surface covering the entire wetland areas.

To enhance the performance of the RF model in VCH prediction, a sensitivity analysis was conducted using $R^{2}$ values to optimize the hyperparameters. Within the GEE platform, RF allows for the adjustment of several hyperparameters, including the number of decision trees (nTree), minimum leaf population, number of variables per split, and bag fraction. The sensitivity analysis was initially performed on the Deer Lake pilot site, and the findings were subsequently applied to the remaining pilot sites. The analysis of model accuracy demonstrated the influence of varying hyperparameter values on the RF model’s performance. For instance, adjusting nTree from 100 to 250 resulted in a change in model accuracy from 0.62 to 0.69. However, it was observed that an optimum nTree value of approximately 200 yielded the same accuracy as a larger nTree. Specifically, a negative correlation was observed between $R^{2}$ and the minimum leaf population. Lower minimum leaf populations were found to achieve higher model accuracy, reaching up to 0.61. On the other hand, the variables per split and bag fraction parameters demonstrated positive correlations with $R^{2}$. Increasing the number of variables per split and incorporating larger fractions of samples for training the model improved the accuracy. The optimal values for these two parameters were determined to be 15 and 0.8, respectively.

### 3.3. Wetland Cover Classification

To create a high-accuracy wetland classification map, the VCH layer was integrated with the feature set mentioned in Section 2. This comprehensive approach equipped the RF classification model with 31 independent variables, optimizing the inputs for wetland mapping. By integrating these features, we aimed to capture the unique surface structure and hydrological characteristics of wetland types for better discrimination. To further explore the classification performance, we also employed the CART (Classification and Regression Trees) method as an alternative approach, and its classification results were compared with those of the RF model.

### 3.4. Accuracy Assessment

The accuracy assessment of VCH maps was conducted by calculating key statistical parameters employing three regression error metrics: Coefficient of determination ($R^{2}$), Root Mean Squared Error (RMSE), and Mean Absolute Error (MAE) based on canopy height reference data from GEDI products. Below are the expressions for these metrics:

(1) $$R^{2}=1-\frac{\sum_{i=1}^{n}{\left(x_{i}-y_{i}\right)}^{2}}{\sum_{i=1}^{n}{\left(y_{i}-\bar{y}\right)}^{2}}$$

(2) $$RMSE=\sqrt{\frac{1}{n}\sum_{i=1}^{n}{\left(x_{i}-y_{i}\right)}^{2}}$$

(3) $$MAE=\frac{1}{n}\sum_{i=1}^{n}\left|x_{i}-y_{i}\right|,$$

where the variables *x*, *y*, and $\bar{y}$ represent the modeled canopy height, reference values, and the average of reference values, respectively.

Additionally, the accuracy of classification maps was assessed using widely recognized metrics: Overall Accuracy (OA), Kappa Coefficient (KC), and F1-score. The formulas for these metrics are provided below:

(4) $$OA=\frac{TP+TN}{TP+FN+FP+TN}$$

(5) $$F1=\frac{2TP}{2TP+FP+FN}$$

(6) $$\begin{array}{c} KC=\frac{PCC-P_{e}}{1-P_{e}}, \end{array}$$

(7) $$\begin{array}{c} PCC=\frac{TN+TP}{N}, \end{array}$$

(8) $$\begin{array}{c} P_{e}=\frac{\left(TP+FP)(TP+FN)+(FN+TN)(FP+TN\right)}{N^{2}}, \end{array}$$

where N=TP+FP+TN+FN, with TP/FP denoting true/false positives, and similarly, TN/FN denoting true/false negatives.

## 4. Experimental Results

### 4.1. VCH Maps and Validation

**Figure 4:**
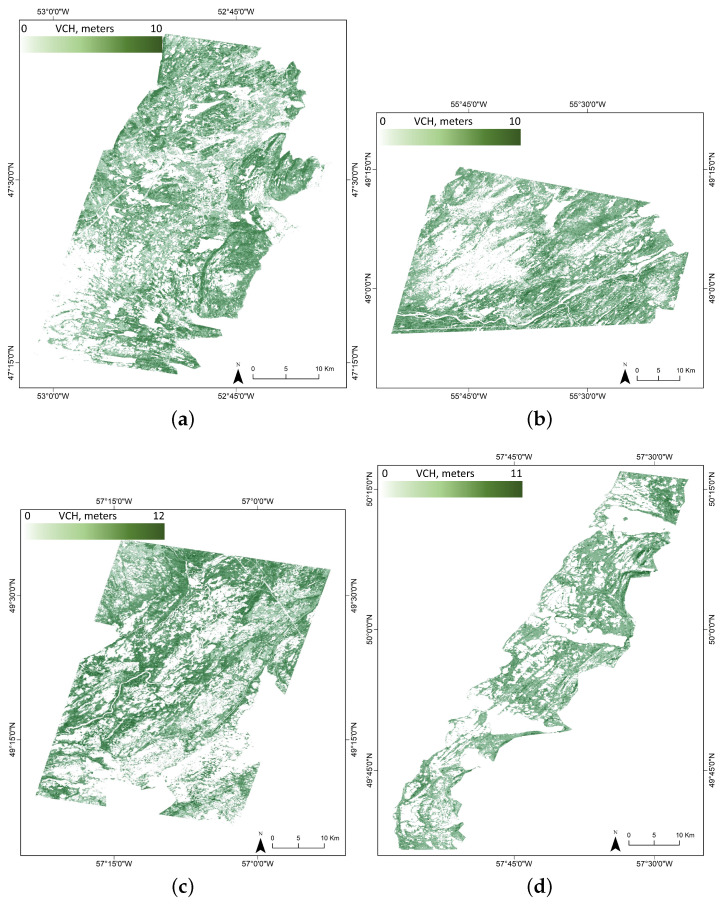
VCH maps for the year 2021 were produced through the integration of GEDI data (June–September 2021) and multi-source EO datasets for (**a**) St. John’s, (**b**) Grand Falls-Windsor, (**c**) Deer Lake, and (**d**) Gros Morne.

As seen in Figure 4, the visual comparison of the generated VCH maps reveals a clear representation of covered wetland height variations. The study areas differ in their characteristics with respect to species composition, site quality (e.g., topography, climate, and hydrology), and slightly varying ranges of canopy heights. The possible inferences for better performance and relatively poor performance of the geostatistical-interpolated VCH maps are discussed below.

**Figure 5:**
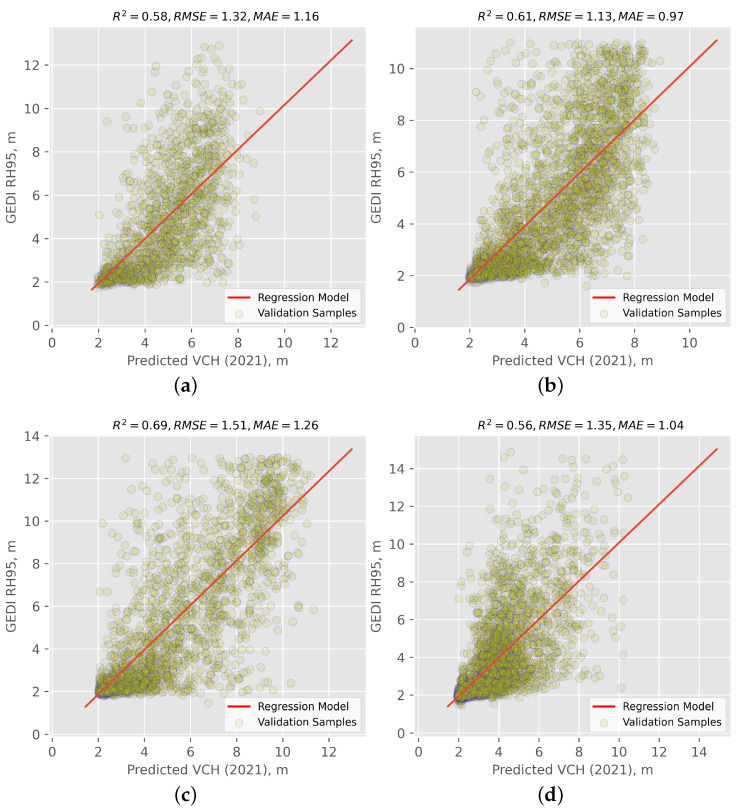
The correlation between the generated VCH and GEDI validation data for the four pilot sites in the study area: (**a**) St. John’s, (**b**) Grand Falls-Windsor, (**c**) Deer Lake, and (**d**) Gros Morne. The figures show the scatter plots of the generated VCH data against the GEDI validation data for each of the four pilot sites. Each plot includes individual data points (dots) and a best-fit line (solid red line) between the two datasets.

To validate the VCH, predictions were compared to the observed and independent testing part of RH95 values for each study site and quantified by deriving statistical measures (i.e., $R^{2}$, RMSE, and MAE). In addition, a scatterplot was drawn in relation to GEDI RH95 data (i.e., observed values) and modeled canopy stand height data (i.e., predicted values) to assess systematic over/under predictions across regions along the range of RH95. The obtained scatter plots with the statistical values are shown in Figure 5.

It is worth mentioning that RH95 does not equate to canopy height. In recent years, several studies have investigated the limitations and uncertainties associated with GEDI observations. For example, refs. [5,51] examined the effect of possible horizontal geolocation inaccuracies and uncertainties on the reliability of GEDI’s forest canopy height estimates. Overall, incorrect ground elevation identification, atmospheric attenuation and thick cloud cover, topography within the footprint, and uncertainty in SNR at the top of the canopy may all result in variations between canopy height estimates and RH95. These factors lead to instability in the accuracy of GEDI observations in complex structure wetland areas. Whereas this study provided good-quality data, some inherent errors remain. Accordingly, even with a high level of model accuracy, the results obtained may still be subject to potential errors. In the following discussion, we will explore these potential errors and their impact on the results drawn from our analysis.

**Figure 6:**
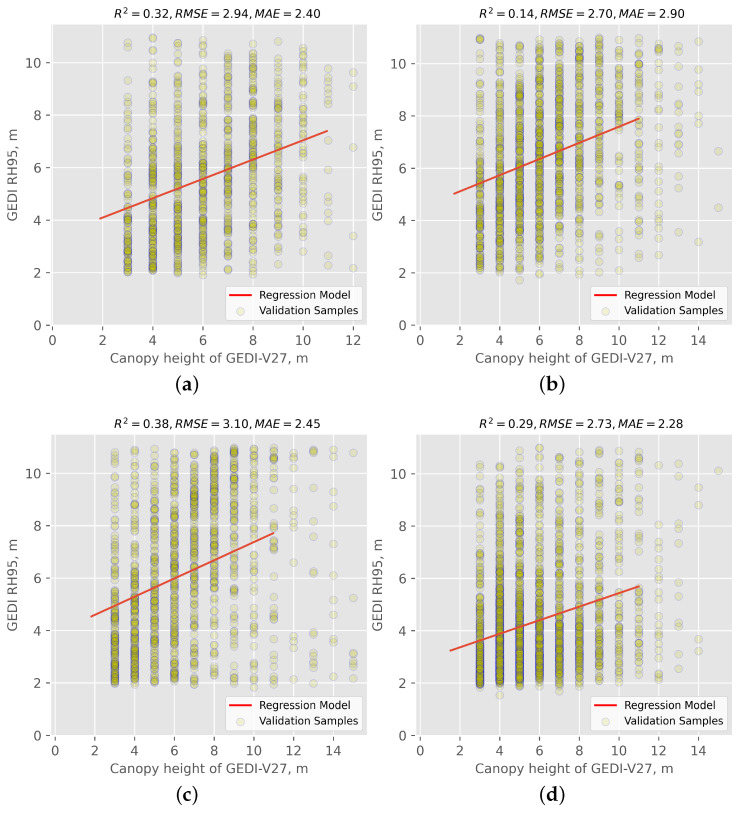
The correlation between the GEDI RH95 observations and GEDI-V27 in wetland areas using the same validation samples of our methodology: (**a**) St. John’s, (**b**) Grand Falls-Windsor, (**c**) Deer Lake, and (**d**) Gros Morne. The figures show the scatter plots of the GEDI-V27 canopy height data against the GEDI validation data for each of the four pilot sites. Each plot includes individual data points (dots) and a best-fit line (solid red line) between the two datasets.

The discrepancy between the 25 m width of GEDI’s observation footprints and the upsampled Sentinel-1 and -2 10 m pixel size can cause issues with straightforward footprint-to-pixel matching. This means that the points in the area observed by GEDI would be sparse when directly matching with the higher resolution imagery from Sentinel-1 and -2; however, vegetation canopy height may vary greatly on adjacent pixels. Because of this, based on the scatterplots in Figure 5, the prediction model is not fully fitted with GEDI measurements. Specifically, neither GEDI footprints nor interpolated canopy height maps can be an exact representation of VCH, and in-situ records are required to decrease the uncertainties and diminish the effect of these technical issues. To explore the potential improvements in generating canopy height maps in this study, the GEDI validation data were compared with the GEDI-V27 global forest canopy height map, which specifically includes only integer values (see Figure 6).

According to Figure 6, it was found that, for all investigated sites, the VCH maps produced in this study exhibit a superior performance to the reference GEDI-V27 data. The limited accuracy of the GEDI-V27 data can be attributed to two primary factors; on the one hand, it is attributed to the bias of geographic positioning, particularly in complex areas with significant elevation variations, which leads to a lower accuracy of the GEDI-V27 product. On the other hand, the GEDI-V27 product was generated based on a global study area; therefore, the accuracy is lower in small-scale test sites. As an example, for the Deer Lake site, the proposed approach produced a VCH map with an $R^{2}$ of 0.69, an RMSE of 1.51 m, and an MAE of 1.26 m (Figure 5) as compared to the GEDI-V27 product with an $R^{2}$ of 0.38, an RMSE of 3.10 m, and an MAE of 2.45 m (Figure 6). Due to the absence of in-situ measurements of canopy height, our study was unable to further investigate the causes for these differences, thereby restricting the progress in increasing the accuracy of the VCH maps.

### 4.2. Wetland Classification Map and Validation

**Figure 7:**
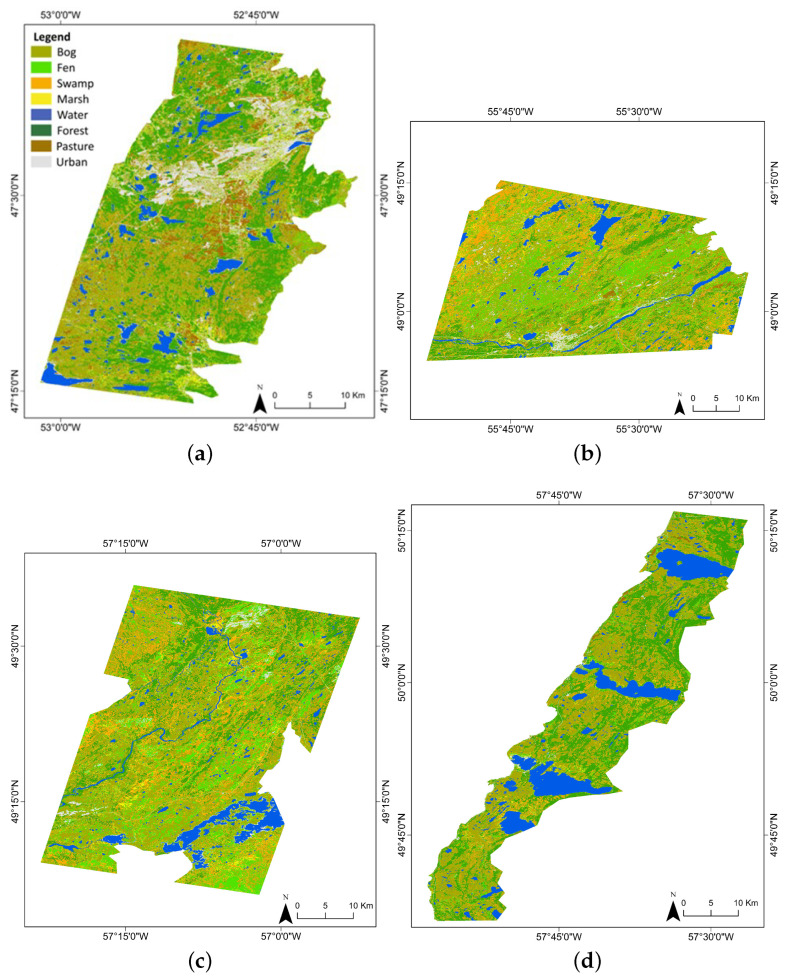
Wetland classification maps for the year 2021 were produced through the integration of VCH and metrics/features derived from multi-source EO datasets for (**a**) St. John’s, (**b**) Grand Falls-Windsor, (**c**) Deer Lake, and (**d**) Gros Morne.

Following the validation of the generated VCH maps with GEDI data, the height layers were stacked with a set of features to provide high-accuracy wetland classification maps. The classification maps illustrating eight wetland and non-wetland covers (i.e., bog, fen, swamp, marsh, deep water, forest, pasture, and urban) are shown in Figure 7.

One of the primary objectives of this study was to assess how well the geostatistical-interpolated VCH from space-borne LiDAR data affects wetland mapping. Initially, the classification was performed with stacked inputs consisting of all variables/features, excluding VCH maps. The results of this initial classification, based on the RF method, yielded an OA range of 82.72% to 88.69% across all wetland sites. The inclusion of the VCH variable derived from GEDI LiDAR increased the number of correctly identified wetland classes, leading to an OA ranging from 86.11% to 93.45%. Adding VCH to the mapping workflow significantly improved the classification results in each area of interest, as evidenced by the comparison between OA values obtained with or without VCH. For instance, at the Deer Lake pilot site, the accuracy increased from 88.69% without VCH to 93.45% with VCH combined with all data sources. This notable improvement in wetland classification demonstrates the importance of integrating GEDI-derived VCH in the mapping process.

Both sets of classification results obtained from the RF and CART models are included in Table 5 for a comprehensive comparison.

**Figure 8:**
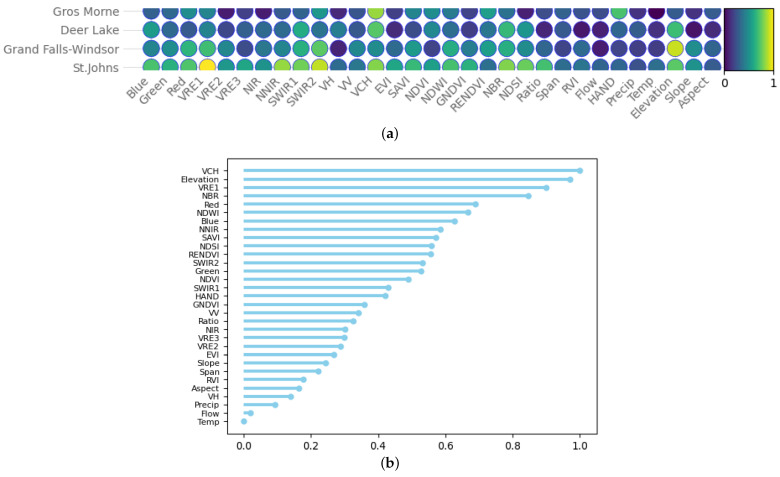
(**a**) The normalized importance of factors used in the wetland classification process of pilot sites, (**b**) the average value of the variables’ importance.

The efficiency of VCH in wetland mapping is further confirmed by investigating the variable importance values in the RF model. In Figure 8, each predictor’s importance value was determined to indicate the most significant features in the wetland classification process. As seen in Figure 8a, there are some general trends for all pilot sites, although some differences exist as well. For instance, the spectral bands of Sentinel-2 show the highest importance for St. John’s and the lowest for Gros Morne pilot sites. Specifically, VCH is among the most significant factors in mapping wetland areas in our study sites. It is ranked in the top three important features along with Elevation and VRE1; see Figure 8b.

In summary, according to validation results, the estimated VCH maps, derived from the integration of multi-source and multi-sensor data, represent a significant advancement in the characterization of wetland ecosystems. The spatially explicit information provided by these maps offers valuable insights into the complex dynamics of vegetation structure and land cover within our study areas. These considerations, in turn, contribute to a deeper understanding of ecosystem dynamics, biodiversity distribution, and land use patterns. Moreover, the integration of VCH maps adds an additional layer of enhancement to wetland mapping, boosting accuracy and reliability.

## 5. Discussion

### 5.1. Leveraging GEDI Data for Large-Scale VCH Mapping

Utilizing laser-based technology, the GEDI mission offers the capability to produce canopy height maps at both high resolution and on a large-scale [52]. This groundbreaking approach provides a multitude of advantages for VCH compared to alternative methods [39]. In the following, some of the key advantages of GEDI data and the opportunities for vegetation studies are highlighted:

- GEDI data yield the 3D structure of the Earth with 25 m diameter footprints [52], ensuring fine spatial resolution for accurately portraying landscape topography and VCH.
- Unlike passive sensor satellite-based methods, GEDI’s laser instrument penetrates dense canopies, providing height measurements even in heavily vegetated regions [53]. This unique capability facilitates the creation of VCH maps as closely as possible to the real world.
- GEDI’s fine spatial resolution, penetration ability through the dense canopies, and information on vertical vegetation structure make it a versatile tool for various applications, particularly large-scale VCH mapping.
- While airborne laser scanning (ALS) data may offer higher accuracy in VCH estimation, they present limitations in terms of coverage and cost. GEDI’s space-borne LiDAR, providing global coverage, emerges as a valuable resource for VCH measurements in regions with limited ALS data availability. Additionally, the cost-effectiveness and consistent dataset provided by GEDI since 2019 eliminate concerns about aligning and matching data from various sources collected at different times. This ensures a standardized approach to estimating VCH.

### 5.2. Leveraging VCH Data Across Environmental Domains

The VCH data play a crucial role in mapping wetlands, especially in complex and densely vegetated environments. In this study, we applied the integration of VCH with other EO data for wetland mapping across four distinct pilot sites in Newfoundland. The utilization of a 10 m GEDI-derived VCH map resulted in an average improvement of ≈5% improvement in OA and ≈0.07 in kappa considering all pilot sites.

This underscores the critical role of VCH as a fundamental tool for wetland mapping, providing valuable information on the structural characteristics of these ecosystems. The synergy achieved by integrating VCH maps with other remote sensing data notably enhances the precision of wetland class discrimination. As wetlands continue to face environmental pressures, the use of VCH, complemented by multi-sensor datasets, represents a robust approach for comprehensive wetland mapping, thereby supporting informed conservation and management strategies.

Beyond enhancing wetland mapping, GEDI measurements of canopy height, canopy vertical structures, and surface elevation have the potential application in various environmental and ecological domains [12,54]:

- Forest structure and aboveground biomass estimation: these measurements provide valuable insights into forest canopy height and structure, enabling accurate estimation of biomass and carbon stocks in forests.
- Land surface dynamics: they can be used to monitor changes in land surface elevation, vegetation dynamics, water cycling processes, and terrain characteristics.
- Biodiversity conservation: these data aid in habitat mapping and characterizing, species distribution modeling, and biodiversity monitoring.

## 6. Conclusions

Accurate wetland mapping is important for effective conservation and restoration planning, as it is vital for monitoring efforts and developing emergency response strategies for natural disasters. This study focused on advancing wetland mapping through the integration of multi-source and multi-sensor remote sensing datasets via GEE. The objective was to introduce complementary EO data and assess their impact on enhancing wetland classification. Exploring GEDI footprints, Merit Hydro, and ERA5 data sources aimed to compensate for the shortcomings associated with relying solely on optical and SAR imagery. Transitioning to an advanced wetland characterization approach, VCH, serving as a representative structural variable, was assessed, and emerged as a key feature for wetland classification in this study. Importantly, optimizing VCH maps and other EO data sources demonstrated their responsiveness to wetland mapping tasks, yielding promising results. The efficiency of VCH was confirmed across all four pilot sites, affirming its effectiveness for wetland studies.

While acknowledging that the VCH maps generated in this study are not the perfect representation of canopy heights, comparative analyses of classification maps indicate progress in achieving high-accuracy wetland maps using VCH. This suggests that the near-to-real state estimation of wetlands’ canopy holds high potential for increasing wetland mapping accuracy.

This conclusion holds true across all study sites, emphasizing the need to extend the research to include the implementation of VCH maps for large-scale wetland mapping (e.g., provincial scale) in future endeavors. Supportive airborne LiDAR data and in-situ measurements are also necessary for a comprehensive assessment and validation of VCH maps.

## Figures and Tables

**Table 1:** Study sites and the geological characteristics.

Pilot Site	Area (km^2^)	Elevation (m)		Vegetation Mean Height (m)	Slope (Degrees)		Land Covers	
		Mean	Max		Mean	Max	Wetland & Non-Wetland	
St. John’s	896	142.33	288	5.88	4.42	74.52	Bog, Fen, Marsh, Swamp,	Water, Forest, Pasture, Urban
Grand Falls-Windsor	1304	135.77	509	5.81	3.23	38.64		
Deer Lake	1241	137.57	455	6.57	3.42	46.80		
Gros Morne	788	61.96	500	6.08	3.87	80.09		

**Table 2:** Number of reference ground-truth polygons for each land cover class.

#	Class	# Polygons
1	Bog	185
2	Fen	185
3	Marsh	150
4	Swamp	157
5	Water	90
6	Forest	184
7	Pasture	155
8	Urban	193

**Table 3:** Partitioning of GEDI Waveforms (N) into training and validation sets.

Pilot Site	N	Train	Test
St. John’s	3266	2286	980
Grand Falls-Windsor	5514	3860	1654
Deer Lake	5786	4050	1736
Gros Morne	5915	4140	1775

**Table 4:** List of predictors used for VCH prediction and wetland cover classification in this study.

Data Source	Band/Variable	Equation	Description
Sentinel-2 Optical	B2 (Blue), B3 (Green), B4 (Red), B5 (VRE1), B6 (VRE2), B7 (VRE3), B8 (NIR), B8A (NNIR), B11 (SWIR1), B12 (SWIR2)	-	Sentinel-2 multispectral bands
Spectral Indices	NDVI	$\frac{NIR-Red}{NIR+Red}$	Normalized Difference Vegetation Index
	SAVI	$1.5\times \frac{\left(NIR-Red\right)}{NIR+Red+0.5}$	Soil Adjusted Vegetation Index
	EVI	$2\times \frac{\left(NIR-Red\right)}{NIR+6\times Red-7.5\times Blue+1}$	Enhanced Vegetation Index
	NDWI	$\frac{Green-NIR}{Green+NIR}$	Normalized Difference Water Index
	GNDVI	$\frac{NIR-Green}{NIR+Green}$	Green Normalized Difference Vegetation Index
	RENDVI	$\frac{NNIR-VRE2}{NNIR+VRE2}$	Red Edge Normalized Difference Vegetation Index
	NBR	$\frac{NIR-SWIR2}{NIR+SWIR2}$	Normalized Burn Ratio
	NDSI	$\frac{Green-SWIR1}{Green+SWIR1}$	Normalized Difference Snow Index
Sentinel-1 SAR	VH, VV	-	Sentinel-1 linear Polarizations
	Span	${\left|VV\right|}^{2}+{\left|VH\right|}^{2}$	Sentinel-1 total scattered power
	Ratio	$\frac{VV}{VH}$	Sentinel-1 cross-polarization ratio
	RVI	$\frac{VH\times 4}{VH+VV}$	Radar Vegetation Index
GEDI	RH95	-	Relative Height
MERIT Hydro- Hydrography	Flow	$F_{out}$ = $F_{in}$ + $F_{local}$	Flow accumulation, where $F_{in}$ is flow received and $F_{local}$ is flow produced
	HAND	$H_{point}$−$H_{drainage}$	Height Above Nearest Drainage, where $H_{point}$ is the DEM elevation and $H_{drainage}$ is the drainage elevation
ERA5–Climate	Precip	-	Average monthly precipitation
	Temp	-	Average monthly temperature
SRTM Topographic DEM	Elevation	-	Height of elevation
	Slope	$\sqrt{D_{x}^{2}+D_{y}^{2}}$	Rate of change of elevation, where $D_{x}$ is horizontal distance and $D_{y}$ is vertical distance
	Aspect	$\arctan\left(\frac{dy}{dx}\right)$	Direction of slope

**Table 5:** Validation results for RF and CART models.

Pilot Site	Method	VCH Included			VCH Excluded		
		OA (%)	Kappa	F1-Score	OA (%)	Kappa	F1-Score
St. John’s	RF	91.66	0.89	0.86	86.22	0.82	0.72
	CART	88.09	0.83	0.83	82.87	0.79	0.7
Grand Falls-Windsor	RF	86.11	0.83	0.81	84.8	0.81	0.77
	CART	84.8	0.82	0.8	79.62	0.76	0.75
Deer Lake	RF	93.45	0.92	0.88	88.69	0.86	0.81
	CART	88.7	0.86	0.84	85.21	0.83	0.79
Gros Morne	RF	90.91	0.85	0.84	82.72	0.73	0.71
	CART	84.21	0.8	0.75	78.88	0.72	0.67

## Data Availability

The code and data are available upon request.
